# Microvolt T-wave alternans at the end of surgery is associated with postoperative mortality in cardiac surgery patients

**DOI:** 10.1038/s41598-019-53760-8

**Published:** 2019-11-22

**Authors:** Chang-Hoon Koo, Hyung-Chul Lee, Tae Kyong Kim, Youn Joung Cho, Karam Nam, Eue-Keun Choi, Sheung-Nyoung Choi, Sehee Yoon, Yunseok Jeon

**Affiliations:** 1 Department of Anaesthesiology and Pain medicine, Seoul National University College of Medicine, Seoul National University Hospital, 101 Daehak-ro, Jongno-gu, Seoul, 03080 Republic of Korea; 2 Present Address: Department of Anaesthesiology and Pain medicine, Seoul National University College of Medicine, Seoul National University Bundang Hospital, 82 Gumi-ro 173 beon-gil, Bundang-gu, Seongnam, 13620 Republic of Korea; 3 Present Address: Department of Anaesthesiology and Pain medicine, Seoul National University College of Medicine, SMG-SNU Boramae Medical Center, 20 Boramae-ro 5-gil, Dongjak-gu, Seoul, 07061 Republic of Korea; 4 Department of Internal Medicine, Seoul National University Hospital, Daehak-ro 101, Jongno-gu, Seoul, 03080 Republic of Korea

**Keywords:** Cardiology, Risk factors

## Abstract

Microvolt T-wave alternans (MTWA), which reflects electrical dispersion of repolarization, is known to be associated with arrhythmia or sudden cardiac death in high risk patients. In this study we investigated the relationship between MTWA and postoperative mortality in 330 cardiac surgery patients. Electrocardiogram, official national data and electric chart were analysed to provide in-hospital and mid-term outcome. MTWA at the end of surgery was significantly associated with in-hospital mortality in both univariate analysis (OR = 27.378, 95% CI 5.616–133.466, p < 0.001) and multivariate analysis (OR = 59.225, 95% CI 6.061–578.748, p < 0.001). Cox proportional hazards model revealed MTWA at the end of surgery was independently associated with mid-term mortality (HR = 4.337, 95% CI 1.594–11.795). The area under the curve of the model evaluating MTWA at the end of surgery was 0.764 (95% CI, 0.715–0.809) and it increased to 0.929 (95% CI, 0.896–0.954) when combined with the EuroSCORE II. MTWA positive at the end of surgery had a 60-fold increase in in-hospital mortality and a 4-fold increase in mid-term mortality. Moreover, MTWA at the end of surgery could predict in-hospital mortality and this predictability is more robust when combined with the EuroSCORE II.

## Introduction

Cardiac surgery is a high-risk procedure with a mortality rate of 2–6%^1^. Therefore, mortality prediction is important to optimise individualised care of cardiac surgery patients. Currently, several tools are available for risk evaluation in such patients^2–4^. Among these tools, the European System for Cardiac Operative Risk Evaluation II (EuroSCORE II) has recently been revised and validated^3^.

Microvolt T wave alternans (MTWA), which is a beat-to-beat alternation of the T wave amplitude, can be easily calculated using electrocardiogram (ECG), a simple, non-invasive and common monitoring procedure. MTWA arises from spatiotemporal heterogeneity of myocardial repolarization, which is an important mechanism of re-entrant arrhythmia^5^. Recent studies have demonstrated that MTWA is associated with arrhythmia or sudden cardiac death in several high-risk patient groups^6–9^. However, the relationship between MTWA and mortality in patients undergoing cardiac surgery has not been evaluated yet.

Therefore, we hypothesised that intraoperative MTWA can predict mortality in patients undergoing cardiac surgery. To evaluate our hypothesis, we conducted a single center study in which the relationship between intraoperative MTWA and mortality was analysed in patients underwent cardiac surgery.

## Methods

### Study design

This study was initially designed as a retrospective analysis using the heart surgery registry at Seoul National University Hospital. This registry enrolled all patients undergoing cardiac and thoracic aortic surgeries at our institution from January 2013 to May 2014. Only patients who refused to participate were excluded and the written informed contents were provided by all these patients. The study was approved by the Institutional Review Board of the Seoul National University Hospital, Seoul, Korea (IRB #1207-111-419) and registered at ClinicalTrial.gov (NCT 01713192). The registry collected perioperative data on intraoperative hemodynamics including intraoperative ECG data, vascular occlusion test using tissue oximetry, temperature, cerebral oximetry, and clinical outcome in patients undergoing cardiac surgery at Seoul National University Hospital from January 2013 to May 2014^10^. The MTWA analysis was additionally approved by the Institutional Review Board of Seoul National University Hospital (IRB #1512-045-727). After analysis of this data, our statistical department recommended that we should enroll a larger number of patients because of the small number of mortality cases in the registry. In retrospective analysis, 4 patients (3.7%, 4/109) died during hospitalization. Previous study recommended that one predictive value could be studied for every 5 events^11^. Assuming that 2–3 variables are considered, we needed 270–405 patients to achieve statistical power.

**Figure 1:**
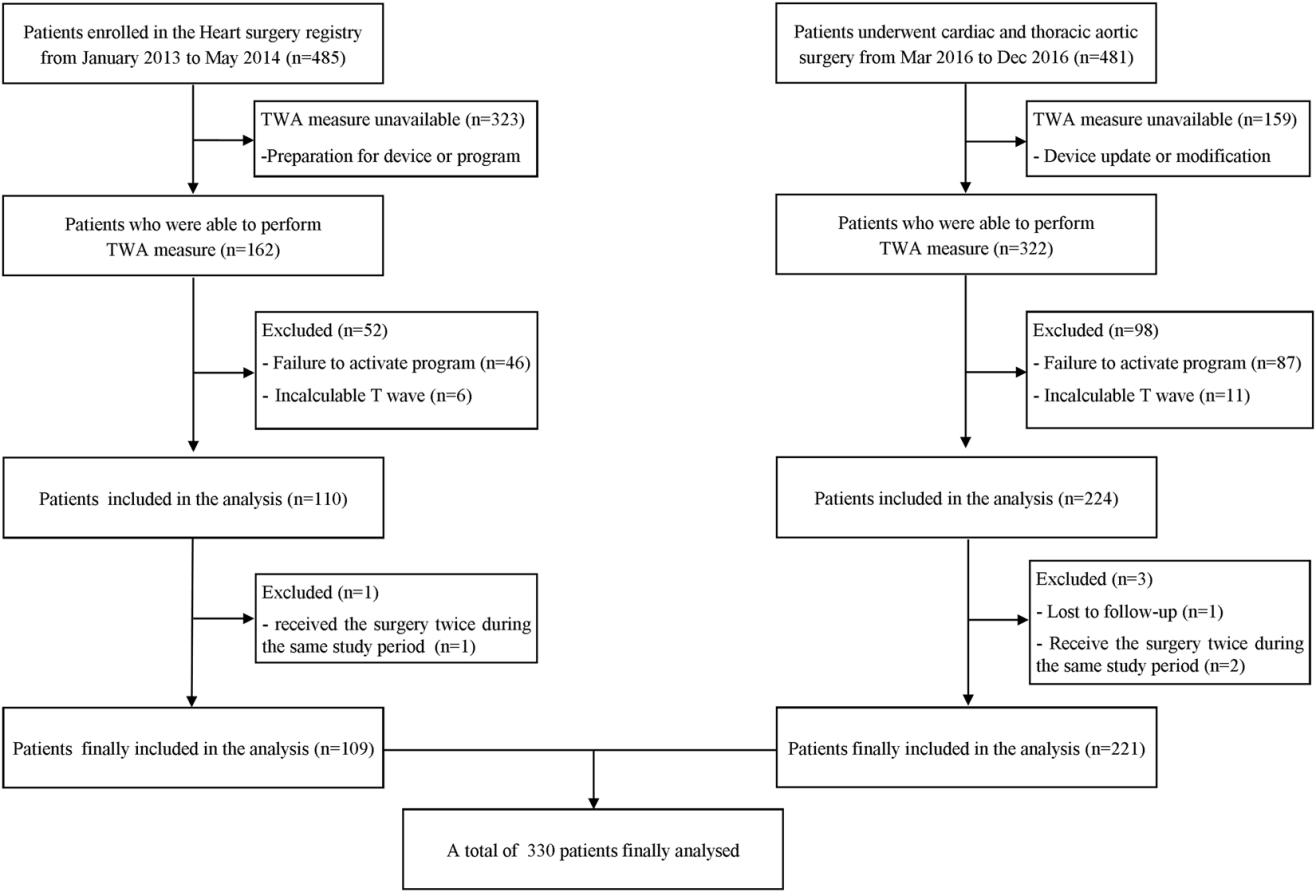
CONSORT diagram. Patients who underwent cardiovascular surgery were enrolled from registry (n = 485) and prospective observational study (n = 481). TWA measurement was available in 162 patients and 322 patients. MTWA calculations were possible in 109 patients and 221 patients. Therefore, a total of 330 patients were finally analysed. TWA = T-wave alternans; MTWA = Microvolt T-wave alternans.

To recruit additional subjects, we performed a prospective observational study from March 2016 to December 2016. This additional recruitment was also approved by the Institutional Review Board of the Seoul National University Hospital, Seoul, Korea (IRB #1602-035-739) and registered at ClinicalTrials.gov (NCT 03201289). Similar to the registry study, all patients undergoing cardiac and thoracic aortic surgeries were enrolled. In this additional prospective observational study, the informed consents were waived by the institutional review board. Thus, ECG data were obtained in all patients except when there were technical problems (Fig. 1). This study was conducted according to the relevant guidelines and national regulations.

### Anaesthesia and intraoperative care

All patients were monitored with ECG, bispectral index (A-2000 XP; Aspect medical Systems, Newton, MA, USA), radial artery catheter, cerebral oximeter, pulmonary artery catheter and transoesophageal echocardiography. Without premedication, anaesthesia was induced with midazolam, sufentanil, and vecuronium. Total intravenous anaesthesia with propofol and remifentanil was used for maintanence of anaesthesia using target controlled infusion. Vecuronium was administered continuously for neuromuscular blockade.

Intraoperative care was performed according to our institution protocol. Attending anaesthesiologists tried to maintain a mean arterial pressure of 60–80 mmHg, a cardiac index of more than 2.0 L/min, and a mixed venous oxygen saturation of more than 60%.

### MTWA testing

When patients arrived at the operating room without premedication, 5-lead ECG monitoring was initiated. Single-lead II ECG signals were digitally recorded at 1000 Hz and 10 bit resolution using a commercial data acquisition system (DI-155, DATAQ Instruments, Inc., Akron, OH). MTWA was analysed using the ECG signals obtained both before anesthesia induction and at the end of surgery using custom software developed in Python v 3.6.1 (Python Software Foundation, Beaverton, OR)^12^. For preprocessing, digitised ECG signals were down-sampled to 500 Hz, and filtered (0.01–100 Hz). MTWA ratio was calculated according the method which was described in the previous studies^13–15^. Baseline wander in ECG was removed by cubic spline interpolation^16,17^. In each clinical situation, 5 minutes of ECG recordings were analysed. From the beginning of the signal, the MTWA ratio was measured using a spectral analysis technique with 128-beat segment window^16^. The beat alignment was performed by maximising the cross correlation of the QRS complex with the template (the average beats in that segment) and equalising the R-peak voltage. When the cross correlation of the QRS complex was <0.95, the beat was rejected and replaced with the template. The window was moved across the 5-minutes ECG recording using a step size of 32 heart beats. The MTWA ratio was calculated for all ECG recordings and was considered positive when the mean MTWA ratio was >3.0^18–20^. The segment was excluded when more than 20% of beats were rejected, or when the heart rate was changed more than 20 beats/min within the segment. Detailed elements of MTWA calculations are described in the Supplementary Material and the source code is available at http://github.com/vitaldb/pyvital/blob/master/ecg _mtwa.py. To evaluate the accuracy of the algorithm implementation, a verification was performed using open database (PhysioNet/Computer in Cardiology Challenge 2008 database)^21^. Fisher’s exact test was conducted to determine whether our implementation can distinguish the synthetic ECG with MTWA from other recordings. In addition, are under receiver characteristic curve (ROC) for detecting synthetic ECG was calculated. The rankings of calculated MTWA ratio were compared to the published data by Kendall’s tau coefficient. The analysis of MTWA results was conducted by H.C. and S.N who were blinded to patients’ characteristics and clinical outcomes.

### Outcomes

The patients were followed up for postoperative period. A review of electric charts was performed through April 2017 by C.H. and S.H. who were blinded to the MTWA results. The mortality data were also confirmed by the official national data of the Ministry of the Interior and Safety on April 2017. The median follow-up period was 1.3 years (interquartile range 0.9–3.4). The primary end point was in-hospital mortality. The other analysed outcomes included mid-term mortality, myocardial infarction, ventricular tachycardia, atrial fibrillation, cardiogenic shock, stroke, renal failure, respiratory failure, and lengths of stay in the intensive care unit and hospital. Myocardial infarction was defined according to the Third Universal Definition of Myocardial Infarction^22^. Details are located in the Supplementary Material. Cardiogenic shock was defined as the need for two or more inotropes for at least 24 hours or the use of a mechanical assist device. Stroke was defined as a new focal neurologic deficit by clinical assessment and brain images of ischemia or hemorrhage lasting >24 hours. Renal failure was defined according to KDIGO criteria 3^23^, which are also shown in the Supplementary Material. Respiratory failure was defined as the need for invasive mechanical ventilation for >48 h.

EuroSCORE II was also calculated for each patient based on electric charts using the formulas available on the EuroSCORE website (http://www.euroscore.org/calc.html).

### Statistical analysis

Continuous variables are shown as medians (interquartile range) and categorical variables as numbers (percentage). The Mann-Whitney U test and Fisher’s exact test were performed to compare baseline characteristics between MTWA-positive and -negative patients. Fisher’s exact test was performed to compare postoperative adverse outcomes between MTWA-positive and -negative patients.

For each patient, the MTWA test results before anesthesia induction and at the end of surgery were compared using the Wilcoxon signed rank test. Logistic regression analysis was performed to identify predictors of in-hospital mortality and to evaluate whether MTWA was independently associated with in-hospital mortality. First, univariate logistic regression analyses were used to identify risk factors for mortality. Subsequently, variables with a p-value < 0.2 in univariate analysis were included in the multivariate analysis and selected according to a forward selection step. Multicollinearity was assessed by variance inflation factor.

Univariate and multivariate Cox proportional hazard regression models (assumptions were checked by Schoenfeld residual plot) were used to determine whether MTWA was associated with mid-term survival. All variables included in the logistic regression model were included in the Cox regression model.

For survival analysis, patients were categorised according to MTWA and the EuroSCORE II. Kaplan-Meier method, log-rank test and multivariate Cox regression analysis were used to evaluate mid-term survival in each group.

A p-value < 0.05 was considered statistically significant and Bonferrnoi correction was used for multiple comparisons. Statistical analyses were performed using SPSS 24 (SPSS Inc., Chicago, IL, USA).

## Results

### Characteristics and number of enrolled patients

In this study, we analysed both registry data and additional prospective observational data as mentioned previously. In the registry, 485 patients underwent cardiovascular surgery from January 2013 to May 2014. MTWA calculations were available at both time points in 109 of 485 patients, and these patients were included in the analysis (Fig. 1). When collecting subsequent prospective observational data, 481 patients underwent cardiac or thoracic aortic surgery from March 2016 to December 2016. Among these patients, MTWA calculations were possible in 221. Therefore, the total number of patients from both the registry and prospective observation study was 330 (Fig. 1). Four patients underwent cardiac surgery twice during the study period. For these patients, the mid-term events were evaluated based on the second surgery. If the MTWA was positive at least one surgery, the patient was considered MTWA positive.

**Figure 2:**
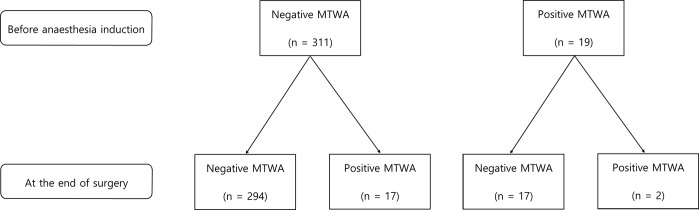
Flowchart of MTWA. 311 patients showed negative MTWA before indcution of anaesthesia. Among them, 17 patients showed positive MTWA at the end of surgery. 19 patients showed positive MTWA before anaesthesia induction, and 2 patients persisted positive MTWA at the end of surgery. MTWA = microvot T-wave alternans.

**Figure 3:**
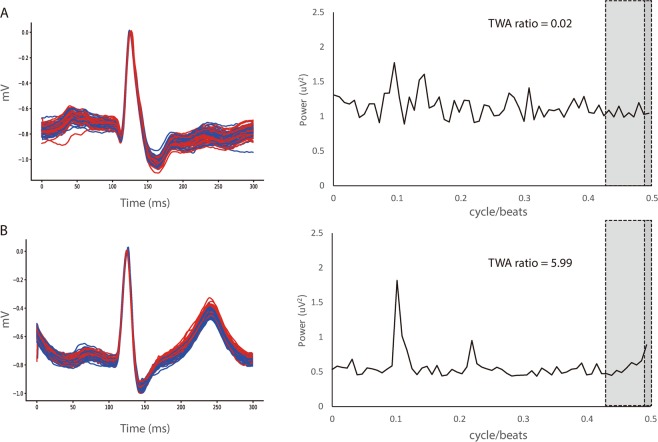
Examples of negative (**A**) and positive (**B**) MTWA at the end of surgery. Even and odd beats are represented by red and blue lines, respectively. MTWA = microvolt T-wave alternans.

MTWA was calculated in 330 patient both before anesthesia induction and at the end of surgery in 330 patients (Fig. 2). MTWA positive rates were 5.8% (19/330) at these two time points, respectively (Fig. 2). Examples of negative and positive MTWA at the end of surgery were depicted in Fig. 3. In the verification of the algorithm using PhysioNet data, our implementation could distinguish synthetic ECG with MTWA from other recordings (P < 0.001, Area under ROC curve [AUC] = 0.863, sensitivity = 37%, specificity = 97% for threshold TWA ratio 3.0). The Kendall’s tau coefficient between the published rankings and those by our implementation was 0.421 (P < 0.001).

The baseline patient characteristics are shown in Table 1. There were no differences in characteristics between MTWA-positive and –negative patients before anesthesia induction, except that MTWA-positive patients used more diuretics and were more likely to undergo mitral valve replacement surgery. In addition, there were no differences between MTWA-positive and –negative patient at the end of surgery, except that patients with positive MTWA underwent aortic arch replacement surgery more frequently (Table 1).

**Table 1:** Baseline characteristics according to T-wave alternans test results.

	Before anaesthesia induction			At the end of surgery		
	Negative (n = 311)	Positive (n = 19)	p-value	Negative (n = 311)	Positive (n = 19)	p-value
Gender(female)	121 (38.9)	9 (47.4)	0.464	123 (39.5)	7 (36.8)	0.815
Age(years)	64 (55–72)	68 (56–75)	0.362	65 (55–73)	56 (50–67)	0.082
Weight(kg)	62.6 (55.9–71.2)	63.3 (49.7–71.1)	0.503	62.5 (55.1–71.1)	63.9 (59.4–76.3)	0.322
Height(cm)	163.2	162.8	0.629	163.2	162.0	0.952
	(156.2–169.0)	(149.3–171.0)		(156.0–169.2)	(157.6–168.7)	
BMI	23.9 (21.7–25.9)	23.4 (20.8–25.6)	0.501	23.8 (21.4–25.9)	24.7 (23.0–25.6)	0.345
**Cormobidities**						
Hypertension	168 (54.0)	12 (63.2)	0.437	168 (54.0)	12 (63.2)	0.437
Diabetes	78 (25.1)	2 (10.5)	0.179	78 (25.1)	2 (10.5)	0.179
CVA	40 (12.9)	2 (10.5)	0.767	40 (12.9)	2 (10.5)	0.767
Arrhythmia	21 (6.8)	1 (5.3)	1.000	19 (6.1)	3 (15.8)	0.124
Angina	33 (10.6)	0	0.236	30 (9.6)	3 (15.8)	0.386
MI	11 (3.5)	0	1.000	10 (3.2)	1 (5.3)	0.485
CHF	21 (6.8)	3 (15.8)	0.141	22 (7.1)	2 (10.5)	0.574
Cardiomyopathy	41 (13.2)	3 (15.8)	0.728	41 (13.2)	3 (15.8)	0.728
Pulmonary disease	29 (9.3)	1 (5.3)	1.000	28 (9.0)	2 (10.5)	0.687
CKD	17 (5.5)	3 (15.8)	0.099	19 (6.1)	1 (5.3)	1.000
Hemodialysis	4 (1.3)	1 (5.3)	0.258	5 (1.6)	0	1.000
**Medications**						
BB	86 (27.7)	6 (31.6)	0.718	85 (27.4)	7 (36.8)	0.374
CCB	50 (16.1)	4 (21.1)	0.529	52 (16.7)	2 (10.5)	0.750
Diuretics	70 (22.5)	10 (52.6)	0.010*	77 (24.8)	3 (15.8)	0.581
ARB	71 (22.8)	4 (21.1)	1.000	73 (23.5)	2 (10.5)	0.264
ACEi	15 (4.8)	1 (5.3)	1.000	16 (5.1)	0	0.611
Statin	40 (12.9)	5 (26.3)	0.157	42 (13.5)	3 (15.8)	0.732
Insulin	17 (5.5)	0	0.611	17 (5.5)	0	0.611
OHA	24 (7.7)	0	0.380	23 (7.4)	1 (5.3)	1.000
Anti-platelet agent	143 (46.0)	12 (63.2)	0.145	147 (47.3)	8 (42.1)	0.662
Smoking	53 (17.1)	3 (15.8)	1.000	52 (16.8)	4 (21.1)	0.544
**ASA class**						
1	6 (1.9)	0	1.000	6 (1.9)	0	1.000
2	106 (34.1)	5 (26.3)	0.487	107 (34.4)	4 (21.1)	0.232
3	189 (60.8)	14 (73.7)	0.261	190 (61.1)	13 (68.4)	0.524
4	10 (3.2)	0	1.000	8 (2.6)	2 (10.5)	0.107
**NYHA class**						
I	107 (34.4)	5 (26.3)	0.470	105 (33.8)	7 (36.8)	0.783
II	133 (42.8)	14 (73.7)	0.008*	136 (43.7)	11 (57.9)	0.228
III	52 (16.7)	0	0.053	52 (16.7)	0	0.053
IV	19 (6.1)	0	0.614	18 (5.8)	1 (5.3)	1.000
Preoperative LVEF	58 (54–64)	61 (50–69)	0.567	58 (54–64)	56 (53–62)	0.227
**Operation types**						
Aortic valve replacement	44 (14.1)	5 (26.3)	0.177	48 (15.4)	1 (5.3)	0.329
Mitral valve replacement	38 (12.2)	7 (36.8)	0.008*	41 (13.2)	4 (21.1)	0.308
Mitral valve replacement with tricuspid annuloplasty	7 (2.3)	0	1.000	6 (1.9)	1 (5.3)	0.342
Mitral valvuloplasty	1 (0.3)	0	1.000	1 (0.3)	0	1.000
Double valve replacement	12 (3.9)	0	1.000	12 (3.9)	0	1.000
Tricuspid valve replacement	8 (2.6)	1 (5.3)	0.418	9 (2.9)	0	1.000
**Tricuspid annuloplasty**						
Pulmonary valve replacement	5 (1.6)	0	1.000	5 (1.6)	0	1.000
Ascending aorta replacement	10 (3.2)	0	1.000	8 (2.6)	2 (10.5)	0.107
Aortic arch replacement	5 (1.6)	0	1.000	3 (1.0)	2 (10.5)	0.028*
Ascending aorta replacement with arch replacement	7 (2.3)	0	1.000	7 (2.3)	0	1.000
Aortic surgery with valve replacement	15 (4.8)	1 (5.3)	1.000	14 (4.5)	2 (10.5)	0.233
Descending thoracic aorta replacement	4 (1.2)	0	1.000	4 (1.2)	0	1.000
Coronary artery bypass graft (off-pump)	104 (33.4)	5 (26.3)	0.522	104 (33.4)	5 (26.3)	0.522
Coronary artery bypass graft (on-pump)	4 (1.2)	0	1.000	3 (1.0)	1 (5.3)	0.212
Mass excision	13 (4.2)	0	1.000	12 (3.9)	1 (5.3)	0.544
Other procedures	31 (10.0)	0	0.236	31 (10.0)	0	0.236
Transplantation	2 (0.6)	0	1.000	2(0.6)	0	1.000
Re-operation	24 (7.7)	2 (10.5)	0.653	25 (8.0)	1 (5.3)	1.000
Emergency	12 (3.9)	2 (10.5)	0.189	14 (4.5)	0	1.000
Op time (min)	360 (310–416.25)	365 (310–433)	0.569	360 (310–415)	365 (300–433)	0.822
Anaesthesia time(min)	435 (380–490)	455 (380–505)	0.470	435 (380–490)	435 (370–495)	0.892

Data are represented as the number (%) or median (interquatile range). BMI = body mass index; CVA = cerebrovascular accident; MI = myocardial infarction; CHF = congestive heart failure; CKD = chronic kidney disease; BB = beta blocker; CCB = calcium channel blocker; ARB = angiotensin receptor blocker; ACEi = angiotensin converting enzyme inhibitor; OAH = Oral anti-hyperglycemic agents; ASA = American Society of Anesthesiologists; NYHA = New York Heart Association; LVEF = left ventricular ejection fraction. *P < 0.05.

### MTWA before anaesthesia induction

Seven patients (2.1%, 7/330) died during hospitalisation (Table 2). The median follow-up period after surgery was 1.3 years (interquartile range 0.9–3.4 years). A total of 28 patients (8.5%) died during the follow up period. Univariate analysis showed that MTWA before anaesthesia induction was not associated with in-hospital or mid-term mortality (Table 3).

**Table 2:** Microvolt T-wave alternans test results and end points.

	Before anaesthesia induction			At the end of surgery		
	Negative (n = 311)	Positive (n = 19)	p-value	Negative (n = 311)	Positive (n = 19)	p-value
In-hospital death	6 (1.9)	1 (5.3)	0.342	3 (1.0)	4 (21.1)	<0.001*
Mid-term death	25 (8.0)	3 (15.8)	0.211	23 (7.4)	5 (26.3)	0.015*
Myocardial infarction	0	0		0	0	
Arrhythmia	28 (9.0)	3 (15.8)	0.405	30 (9.6)	1 (5.3)	1.000
Ventricular tachycardia	2 (0.6)	0	1.000	2 (0.6)	0	1.000
New onset Atrial fibrillation	49 (15.8)	3 (15.8)	1.000	49 (15.8)	3 (15.8)	1.000
Cardiogenic shock	21 (6.8)	2 (10.5)	0.632	19 (6.1)	4 (21.1)	0.035*
Stroke	2 (0.6)	0	1.000	1 (0.3)	1 (5.3)	0.112
Renal failure	13 (4.2)	1 (5.3)	0.572	12 (3.9)	2 (10.5)	0.189
Respiratory failure	20 (6.4)	2 (10.5)	0.367	19 (6.1)	3 (15.8)	0.124
ICU stay (days)	2 (1–3)	2 (1–3)	0.309	3 (2–4)	2 (1–6)	0.712
Hospital stay (days)	10 (8–14)	11 (9–15)	0.958	14 (10–21)	14(11–21)	0.704

Data are represented as the number (%) or median (interquartile range). ICU = intensive care unit.

*P < 0.05.

**Table 3:** Univariate and Multivariate Logistic Regression Analysis of Risk Factors for In-hospital mortality.

Variable	Univariable Analysis		Multivariable Analysis	
	OR (95% CI)	p-value	OR (95% CI)	p-value
**MTWA**				
MTWA before induction	2.824 (0.323–24.727)	0.348		
MTWA at the end of surgery	27.378 (5.616–133.466)	<0.001*	59.375 (6.075–580.274)	<0.001*
**Demographic data**				
Gender	0.864 (0.190–3.925)	0.850		
Age > 75	0.910 (0.107–7.722)	0.931		
BMI > 30 kg/m^2^		0.999		
**Medical history**				
ASA class III/IV	3.362 (0.400–28.270)	0.264		
NYHA class III/IV	1.472 (0.280–7.754)	0.648		
Hypertension	1.114 (0.245–5.056)	0.889		
Diabetes		0.997		
CVA		0.998		
Arrhythmia	6.060 (1.106–33.209)	0.038		
Angina	1.516 (0.177–12.989)	0.704		
CHF	20.200 (4.228–96.498)	<0.001*	44.338 (4.567–430.456)	0.001*
Cardiomyopathy	9.433 (2.036–43.701)	0.004*		
COPD	3.422 (0.387–30.259)	0.269		
CKD	6.778 (1.229–37.375)	0.028*		
Hemodialysis	13.292 (1.286–137.351)	0.030*		
Smoking	0.809 (0.095–6.855)	0.846		
**Preoperative medication**				
BB	0.423 (0.05–3.563)	0.429		
CCB		0.997		
Diuretics	4.333 (0.949–19.790)	0.058		
ARB	0.561 (0.066–4.733)	0.595		
ACEi		0.999		
Statin	2.605 (0.490–13.850)	0.261		
Insulin		0.999		
OAH		0.998		
Anti-platelet agent	2.883 (0.551–15.079)	0.210		
**Preoperative details**				
Preoperative LVEF	0.996 (0.948–1.048)	0.885		
Preoperative LVEF < 40%		0.998		
**Surgery details**				
Duration of surgery	1.008 (1.003–1.012)	0.002*		
previous cardiac surgery	4.983 (0.918–27.054)	0.063		
Emergency		0.999		
**Anesthesia details**				
Duration of anaesthesia	1.007 (1.003–1.012)	0.002*		
Intraoperative epinephrine	3.198 (0.363–28.172)	0.295		
Intraoperative norepinephrine	1.020 (0.225–4.631)	0.979		
Intraoperative nitroglycerin	0.276 (0.053–1.446)	0.128		
Intraoperative dobutamine	1.325 (0.292–6.015)	0.715		
Intraoperative dopamine		0.999		
Last MBP	0.918 (0.856–0.984)	0.016*		
Last CVP	1.125 (0.993–1.274)	0.063		
Mechanical assist device	31.795 (6.442–156.927)	<0.001*		
Last Lactate level	0.872 (0.298–2.577)	0.804		
**Postoperative ECG**				
Arrhythmia		0.998		
Ventricular tachycardia		1.000		
New-onset atrial fibrillation		0.997		

OR = odds ratio; CI = confidence interval; MTWA = Microvolt T-wave alternans; BMI = body mass index; ASA = American Society of Anesthesiologists; NYHA = New York Heart Association; CVA = cerebrovascular accident; CHF = congestive heart failure; COPD = chronic obstructive pulmonary disease; CKD = chronic kidney disease; BB = beta blocker; CCB = calcium channel blocker; ARB = angiotensin receptor blocker; ACEi = angiotensin converting enzyme inhibitor; OAH = oral anti-hyperglycemic medications; LVEF = left ventricular ejection fraction; MBP = mean blood pressure; CVP = central venous pressure.

*P < 0.05.

### In-hospital mortality and MTWA at the end of the surgery

Among the seven patients (2.1%, 7/330) who died during hospitalization, four were MTWA-positive at the end of surgery. In three of these four patients, ECMO (extracorporeal membrane oxygenation) was used during the weaning and postoperative periods. One patient among these three patients died from ischemic colitis. The other two patients among these three patients died from multi-organ failure. The last patient who showed MTWA-positive at the end of surgery and died during in-hospital period received hybrid thoracic endovascular aortic repair. This patient was stable except for a wound problem in the inguinal area. However, the patient suddenly expired on 22th postoperative day. An autopsy was not performed, so the cause of death was not clear. Detailed causes of mortality were summarized in Supplementary Table S1.

Mechanical assist devices were used in fifteen patients (4.5%, 15/330) during postoperative period. Among these fifteen patients, five patients showed positive MTWA at the end of surgery in whom three patients died during hospital period. So, in-hospital mortality of the patients with postoperative mechanical assist device and MTWA positive at the end of surgery was 60% (3/5). In the other 10 patients, mechanical assist devices were used, but MTWA was negative at the end of surgery. In-hospital mortality of these patients was 10% (1/10).

The in-hospital mortality was 21.1% (4/19) among the patients who were MTWA positive versus 1.0% (3/311) among those MTWA negative at the end of surgery (p < 0.001). For the other clinical outcomes, cardiogenic shock was more frequent in patients who were MTWA positive at the end of surgery (21.1% versus 6.1%, p = 0.035) (Table 2).

In the univariate regression model, MTWA at the end of surgery was significantly associated with in-hospital mortality (odds ratio [OR] = 27.378, 95% confidence interval [CI] 5.616–133.466, p < 0.001). Other variables with a p < 0.2 in the univariate analysis (MTWA at the end of surgery, arrhythmia, congestive heart failure, cardiomyopathy, chronic kidney disease, hemodialysis, use of diuretics, duration of surgery, intraoperative nitroglycerin infusion, last measured intraoperative mean arterial pressure, last measured intraoperative central venous pressure and application of mechanical assist device) were included in the multivariate analysis and selected according to a forward selection. The maximum variance inflation factor was 1.326, suggesting that multicollinearity was not a problem.

Multivariate analysis revealed that MTWA at the end of surgery (OR = 59.225, 95% CI 6.061–578.748, p < 0.001) and congestive heart failure (OR = 44.226, 95% CI 4.556–429.325, p = 0.001) were independent risk factors for in-hospital mortality.

**Figure 4:**
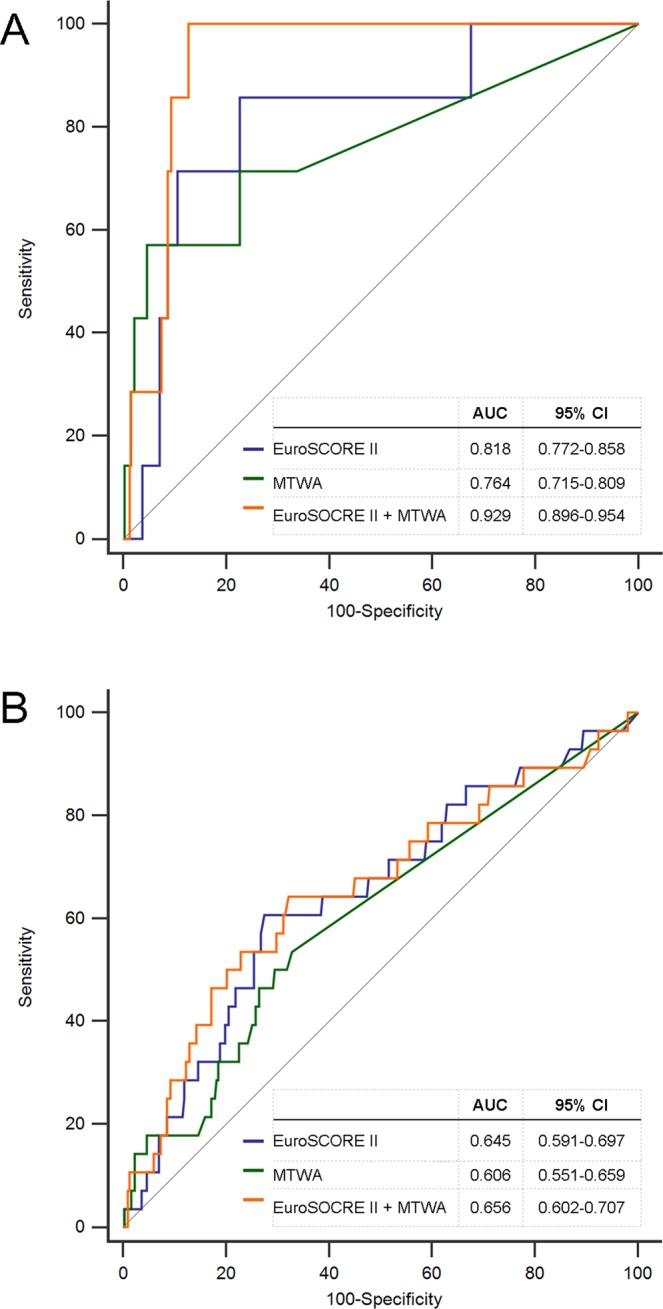
Receiver operating characteristic (ROC) curve. (**A**) in-hospital mortality, The AUC of MTWA was 0.764 (95% CI, 0.715–0.809) compared with 0.818 (95% CI 0.772–0.858) for EuroSCORE II and 0.929 (95% CI 0.896–0.954) for EuroSCORE II + MTWA. (**B**) mid-term mortality. The AUC of MTWA was 0.606 (95% CI, 0.551–0.659) compared with 0.645 (95% CI 0.591–0.679) for EuroSCORE II and 0.656 (95% CI 0.602–0.707) for EuroSCORE II + MTWA. MTWA = microvolt T-wave alternans; AUC = area under curve; CI = confidence interval.

A ROC curve for prediction of in-hospital mortality from MTWA at the end of surgery is shown in Fig. 4. The AUC was 0.764 (95% CI, 0.715–0.809) and it increased to 0.929 (95% CI, 0.896–0.954) when combined with the EuroSCORE II. The AUC of the EuroSCORE II was 0.818 (95% CI, 0.772–0.858). (Fig. 4).

### Mid-term mortality and MTWA at the end of the surgery

The rate of mid-term mortality was significantly higher in patients with positive than those with negative MTWA at the end of surgery (26.3% versus 7.4%, respectively, p = 0.015). In the univariate Cox regression analysis, MTWA at the end of surgery also increased mid-term mortality (HR = 5.485, 95% CI 2.059–14.612, p = 0.001). In multivariate analysis, MTWA at the end of surgery also increased mid-term mortality (HR = 4.337 95% CI 1.594–11.795, p = 0.004). Other variables with a p < 0.2 in the univariate analysis were age >75, ASA class III/IV, diabetes, arrhythmia, congestive heart failure, chronic obstructive pulmonary disease, chronic kidney disease, use of diuretics, use of statin, use of insulin, use of antiplatelet agents, duration of surgery, intraoperative nitroglycerin infusion, last measured intraoperative mean arterial pressure, last measured intraoperative central venous pressure and application of mechanical assist device. Cox proportional hazards model assumptions were satisfied in all these variables^24,25^. Among these variables, age >75, chronic kidney disease, duration of surgery and the use of mechanical assist devices were also independently associated with mid-term mortality (Table 4).

**Table 4:** Univariable and Multivariable Cox Proportional Hazards Regression Analysis for mid-term mortality.

Variable	Univariable Analysis		Multivariable Analysis	
	HR (95% CI)	p-value	HR (95% CI)	p-value
**MTWA**				
MTWA before induction	1.554 (0.467–5.171)	0.472		
MTWA at the end of surgery	5.485 (2.059–14.612)	0.001*	4.337 (1.594–11.795)	0.004*
**Demographic data**				
Gender	1.103 (0.508–2.393)	0.804		
Age > 75	3.461 (1.593–7.521)	0.002*	3.471 (1.475–8.165)	0.004*
BMI > 30 kg/m^2^	0.046 (0.000–107.412)	0.437		
**Medical history**				
ASA class III/IV	2.418 (0.977–5.989)	0.056		
NYHA class III/IV	1.030 (0.387–2.743)	0.952		
Hypertension	1.451 (0.678–3.104)	0.337		
Diabetes	1.737 (0.081–3.764)	0.162		
CVA	0.851 (0.257–2.818)	0.791		
Arrhythmia	2.475 (0.932–6.570)	0.069		
Angina	1.476 (0.350–6.220)	0.596		
CHF	4.036 (1.636–9.961)	0.002*		
Cardiomyopathy	1.509 (0.629–3.620)	0.357		
COPD	3.691 (1.397–9.753)	0.008*		
CKD	7.120 (2.995–16.930)	<0.001*	3.842 (1.502–9.825)	0.005*
Hemodialysis	0.049 (0.000–132663)	0.689		
Smoking	0.706 (0.244–2.042)	0.521		
**Preoperative medication**				
BB	0.624 (0.253–1.542)	0.307		
CCB	0.674 (0.233–1.951)	0.467		
Diuretics	1.688 (0.779–3.659)	0.185		
ARB	1.414 (0.623–3.212)	0.408		
ACEi	0.728 (0.099–5.357)	0.755		
Statin	1.840 (0.805–4.204)	0.148		
Insulin	2.532 (0.874–7.334)	0.087		
OAH	0.044 (0.000–63.784)	0.401		
Anti-platelet agent	1.809 (0.833–3.931)	0.134		
**Preoperative details**				
Preoperative LVEF	1.000 (0.972–1.029)	0.993		
Preoperative LVEF < 40%	0.042 (0.000–9.171)	0.249		
Surgery details				
Duration of surgery	1.004 (1.001–1.007)	0.003*	1.005 (1.002–1.008)	<0.001*
previous cardiac surgery	1.787 (0.535–5.964)	0.345		
Emergency	1.106 (0.149–8.201)	0.922		
**Anesthesia details**				
Duration of anaesthesia	1.004 (1.001–1.007)	0.005*		
Intraoperative epinephrine	2.133 (0.499–9.124)	0.307		
Intraoperative norepinephrine	1.035 (0.434–2.468)	0.939		
Intraoperative nitroglycerin	0.481 (0.220–1.050)	0.066	0.357 (0.152–0.843)	0.019*
Intraoperative dobutamine	1.643 (0.750–3.601)	0.215		
Intraoperative dopamine	1.565 (0.211–11.618)	0.662		
Last MBP	0.977 (0.950–1.005)	0.105		
Last CVP	1.057 (0.989–1.129)	0.103		
Mechanical assist device	7.290 (2.938–18.090)	<0.001*	4.500 (1.764–11.477)	0.002*
Last Lactate level	1.129 (0.666–1.914)	0.653		
**Postoperative ECG**				
Arrhythmia	0.509 (0.120–2.164)	0.360		
Ventricular tachycardia	0.049 (0.000–1941932411)	0.809		
New-onset atrial fibrillation	0.578 (0.174–1.915)	0.370		

HR = hazard ratio; CI = confidence interval; MTWA = Microvolt T-wave alternans; BMI = body mass index; Hx = history; ASA = American Society of Anesthesiologists; NYHA = New York Heart Association; CVA = cerebrovascular accident; CHF = congestive heart failure; COPD = chronic obstructive pulmonary disease; CKD = chronic kidney disease; BB = beta blocker; CCB = calcium channel blocker; ARB = angiotensin receptor blocker; ACEi = angiotensin converting enzyme inhibitor; OAH = oral anti-hyperglycemic medications; LVEF = left ventricular ejection fraction; MBP = mean blood pressure; CVP = central venous pressure. *P < 0.05

The AUCs for MTWA, the EuroSCORE II and both combined for predicting mid-term mortality were 0.606 (95% CI 0.551–0.659), 0.645 (95% CI 0.591–0.697) and 0.656 (95% CI 0.602–0.707), respectively. A EuroSCORE II >3.42 was determined by ROC analysis as the optimal cut-off value to predict mid-term mortality.

**Figure 5:**
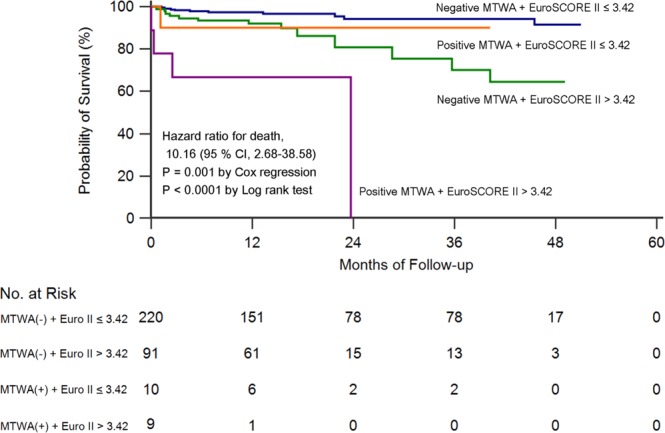
Kaplan-Meier curves for mid-term mortality. Patients were categorized according to MTWA (positive vs. negative) and the EuroSCORE II (≤3.42 vs. >3.42). Group I = Negative MTWA + EuroSCORE II ≤ 3.42, Group II = Negative MTWA + EuroSCORE II > 3.42, Group III = Positive MTWA + EuroSCORE II ≤ 3.42, Group IV = Positive MTWA + EuroSCORE II > 3.42. Mid-term mortality was 4.1% (9/220), 15.4% (14/91), 10% (1/10), and 44.9% (4/9) for Groups I to IV, respectively. MTWA = microvolt T-wave alternans; CI = confidence interval; Euro II = Euroscore II.

Patients were divided into four groups according to MTWA (positive vs. negative) and the EuroSCORE II (≤3.42 vs. >3.42) (Fig. 5). Mid-term mortality of the patient with negative MTWA and a low EuroSCORE II was 4.1% (9/220). On the other hand, mid-term mortality of the patient with positive MTWA with a high EuroSCORE II was 44.4% (4/9). Kaplan-Meier survival method showed that patients with positive MTWA and a high EuroSCORE II (>3.42) had a lower survival rate compared with the patients with negative MTWA and a low (p < 0.001) and negative MTWA and a high EuroSCORE II (p < 0.001) (Fig. 5). In multivariate Cox regression analysis, patients with negative MTWA and a low EuroSCORE II had a higher survival rate compared with those with negative MTWA and a high EuroSCORE II (HR = 2.765, 95% CI 1.099–6.953, p = 0.031) and those with positive MTWA and a high EuroSCORE II (HR = 10.163, 95% CI 2.677–38.583, p = 0.001) (Fig. 5).

## Discussions

In this study, patients with a positive MTWA at the end of surgery had an almost 60-fold higher in-hospital mortality (OR = 59.225) compared with patients with a negative MTWA at the end of surgery. Cardiogenic shock and coma were also more frequent in these patients (3.5 and 5 times, respectively. The mid-term mortality rate was approximately 4-fold higher in patients with positive MTWA at the end of surgery (OR = 4.337) compared with patients with a negative MTWA at the end of surgery. The AUC of the ROC curve to predict in-hospital mortality by MTWA at the end of surgery was 0.764, and this AUC increased to 0.929 when combined with the EuroSCORE II.

Differences in myocardial repolarization appears as MTWA^19^. This alternans are either concordant or discordnat^26^. Discordant alternans could be induced by short repolarization in two consecutive beats either at epi-myocardium or mid-myocardium^26^. First, stepwise shortening of cycle length superimposed dispersion of repolarization could result in different diastolic intervals. Second, if cycle length is suddenly shortened, the dependence of conduction velocity on diastolic intervals could result in different conduction delay, and different diastolic intervals. Different diastolic intervals could induce discordant T wave alternans^26^. MTWA reflects increased repolarization heterogeneity and the risk of ventricular arrhythmia^16,19,27^. Therefore, it can be useful for predicting the risk of cardiac death in high-risk patients and for screening patients who need intensive treatments^28,29^. In addition, MTWA has been found to be related with mortality not only in high-risk patients but also in low risk patients^30^.

MTWA can be a valuable tool for evaluating high-risk patients. Patients with ischemic cardiomyopathy can be screened for risk of sudden cardiac death using MTWA^7,31^. MTWA is an independent predictor of ventricular tachyarrhythmia in patients with dilated cardiomyopathy^32^. There is also was a significant association between positive MTWA and mortality in patients who have left ventricular dysfunction^33^. However, few studies have evaluated the perioperative applications of MTWA.

This study demonstrated that positive MTWA at the end of surgery was significantly associated with serious postoperative complications including in-hospital death, mid-term death, cardiogenic shock and coma. MTWA is affected by various factors including an increased heart rate, ventricular premature beats, coronary artery occlusion and reperfusion, adrenergic stimulation, mental stress and myocardial ischemia^19^. MTWA magnitude may be amplified by myocardial ischemia^19^. Myocardial ischemia or reperfusion injury may commonly occur in cardiovascular surgery, and deterioration of cardiac function may occur after ischemia- reperfusion injury^34^. Therefore, it can be inferred that patients with positive MTWA at the end of surgery had vulnerable myocardial substrates for ischemia or reperfusion injury. However, MTWA before anaesthesia induction did not increase postoperative mortality in this study. Of the 19 patients who were MTWA positive before anaesthesia induction, 17 had negative results at the end of surgery. We therefore assumed that cardiac surgery itself, such as successful revascularization may attenuate the factor which caused MTWA positive before surgery and that may be the reason MTWA before surgery was related with postoperative mortality.

Another independent variable related to in-hospital mortality included congestive heart failure. Duration of surgery, age >75 years, chronic kidney disease, intraoperative nitroglycerin infusion and the use of mechanical assist devices were also independent risk factors of the mid-term mortality.

A previous study identified previous cardiac surgery, emergency operations, cardiac failure, age >75 years, angina CCS class IV, complex surgical procedures, three-vessel coronary disease and female sex as predictors of mortality in patients undergoing cardiac surgery^35^. Many models have been developed to predict postoperative mortality based on combinations of the risk factors that affect mortality after cardiac surgery. Among them, the EuroSCORE II is the most recently proposed and widely used. EuroSORE II is a revised version of the EuroSCORE I^36^. However, a meta-analysis showed that EuroSCORE II still tends to overestimate mortality, predicting a mortality rate of 3.30% compared with the actual mortality rate of 2.95%^37^. This agrees with our results, which showed an actual mortality rate of 2.12% (7/330) and a predicted mortality rate of 3.37% using the EuroSCORE II. The AUC for Eurscore II was 0.792 in that meta-analysis and 0.818 in our study^37^. Unlike in-hospital mortality, the EuroSCORE II had a very low predictive validity as a risk model for mid-term mortality in this study. This can be explained by the fact that in most studies evaluating EuroSCORE II, the endpoints short-term mortality such as 30-day mortality or in-hospital mortality^37^. A trend toward an increased AUC was observed when combining MTWA and the EuroSCORE II and it reached 0.929. Moreover, mid-term mortality of the patient with positive MTWA and a high EuroSCORE II had a 10-fold higher mid-term mortality compared with that of the patient with negative MTWA and a low EuroSCORE II. Therefore, it is proposed that the patients with a high EuroSCORE II and positive MTWA at the end of surgery have very poor clinical prognosis.

Until now, methods of measuring MTWA have not been standardised, and differences in study methods exist. The reproducibility of the MTWA test is also low^38^. In addition, evidence for early interventions, such as implantable cardioverter defibrillation, based on MTWA result is not yet sufficient^6^. Nevertheless, MTWA has a high negative predictive value for cardiac death^31,39^. In addition, because ECG is a standard monitoring technique used during anesthesia, MTWA testing during anesthesia requires no additional interventions. Therefore, a MTWA test can give helpful information to evaluate the risks for patients after cardiac surgery.

There are some limitations to this study. First, though MTWA showed strong relationship with postoperative mortality, it does not suggest what to do for the patients with positive MTWA. Positive MTWA in these cases was just a marker of very sick patients who were in circulatory shock.

Second, the method used in this study has not been fully tested. Although we validated the performance of our implementation using Physionet data, it was not compared with commercialized device such as the HeartWave system (Cambridge Heart Inc., Bedford, MA, USA). However, because we opened it on the public repository, details of our implementation of calculating MTWA and the parameters such as the moving steps, time for averaging, and the threshold value of TWA ratio can be optimized in future studies using our implementation.

Third, the cause and effect relationship between MTWA and in-hospital death is uncertain. Moreover, the causes of mortality in cardiac surgery patients could not only be cardiogenic ones, but also many others such as bleeding or infection. This may explain the low sensitivity of MTWA test in this study. Nevertheless, a MTWA test may give the clinicians useful information since MTWA test showed high specificity in this study. Combined with other parameters, MTWA test could be a practical risk prediction system in cardiac surgical patients.

Fourth, our study results do not suggest any early intervention to decrease postoperative mortality when the patients show positive MTWA at the end of surgery. Most patients with MTWA positive in our study did not show ventricular arrhythmia. Therefore, myocardial ischemia and excessive adrenergic stimulation may be important pathophysiology in these patients. If then, resting the myocardium might be helpful in these MTWA positive patients such as avoiding use of inotropics, and venting the left ventricle during ECMO. However, this topic is beyond our study and we may need further study.

Finally, we enrolled 330 patients in total, which may still be too low to evaluate mortality effectively. There is a concern of relatively small sample size due to over-fitted model. More than half of patients who underwent cardiovascular surgery during study period were excluded due to technical problems such as device malfunction.

To summarize, due to practical limitations, MTWA could only be assessed in a limited subset of patients and the temporal stability of MTWA after surgery could not be evaluated. Additional studies on the robustness of MTWA as predictive marker in a larger cohort are needed.

In conclusion, patients who underwent cardiac surgery and were MTWA positive at the end of surgery had a 60-fold increase in in-hospital mortality and a 4-fold increase in mid-term mortality. MTWA at the end of surgery was able to predict in-hospital mortality, and this predictive ability is more robust when combined with the EurosSCORE II. MTWA at the end of surgery has profound clinical significance in patient undergoing cardiac surgery.

## Supplementary information

Supplementary information file
