# Supplementary material for: Microvolt T-wave alternans at the end of surgery is associated with postoperative mortality in cardiac surgery patients
Source: Sci Rep. 2019 Nov 22;9:17351. doi: 10.1038/s41598-019-53760-8 (PMC6874567; doi:10.1038/s41598-019-53760-8)
Supplement: Supplementary file 1 — Supplementary information file [file 41598_2019_53760_MOESM1_ESM.pdf]

Microvolt T-wave alternans at the end of surgery is associated with postoperative mortality in cardiac surgery patients

Chang-Hoon Koo, M.D.Ph.D.[1,2]#, Hyung-Chul Lee, M.D.,Ph.D.[1]#, Tae Kyong Kim, M.D.,Ph,D.[1,3], Youn Joung Cho, M.D.[1], Karam Nam, M.D.[1], Eue-Keun Choi, M.D.,Ph.D.[4], Sheung-Nyoung Choi, M.D.[1], Sehee Yoon, M.D.[1], Yunseok Jeon, M.D., Ph.D.[1]*

#These two authors equally contributed to this work as co-first authors.

[1]Department of Anaesthesiology and Pain medicine, Seoul National University College of Medicine, Seoul National University Hospital, 101 Daehak-ro, Jongno-gu, Seoul 03080, Republic of Korea

(current position) [2]Department of Anaesthesiology and Pain medicine, Seoul National University College of Medicine, Seoul National University Bundang Hospital, 82 Gumi-ro 173 beon-gil, Bundang-gu, Seongnam 13620, Republic of Korea

(current position) [3]Department of Anaesthesiology and Pain medicine, Seoul National University College of Medicine, SMG-SNU Boramae Medical Center, 20 Boramae-ro 5-gil, Dongjak-gu, Seoul 07061, Republic of Korea

[4]Department of Internal Medicine, Seoul National University Hospital, Daehak-ro 101, Jongno-gu, Seoul 03080, Republic of Korea

Corresponding Author

Yunseok Jeon, MD, PhD

Address: Department of Anaesthesiology and Pain Medicine, Seoul National University Hospital, 101 Daehak-ro, Jongno-gu, Seoul 03080, Republic of Korea

Phone: +82-2-2072-2467

Fax: +82-2-747-5639

Email: jeonyunseok@gmail.com

Supplementary information 1. Detailed description of calculating microvolt T-wave alternans

ECG preprocessing

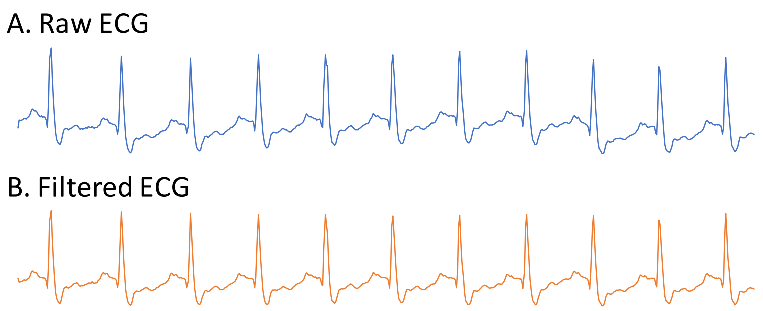

Figure 1. A. Raw ECG from our dataset. B. bandpass filtered (with low cutoff frequency of 0.01Hz and high cutoff frequency of 100Hz) and baseline wander removed (using the cubic-spine algorithm) ECG. ECG=Electrocardiogram.

The raw electrocardiogram (ECG) was bandpass filtered with cutoff frequency of 0.01 and 100 Hz. (Fig. 1) To remove the baseline wander of the ECG a method based on the cubic-spine algorithm was used. [1] At first, threshold-based R-peak detection algorithm was applied. After R-peaks detected, the maximum downslope after R-peak was determined in each beat. The PQ interval 'knot' was placed at 66 milliseconds before the maximum downslope. The cubic spline curves passing all the knots were obtained and subtracted from the original waveform. [2]

ECG segmentation

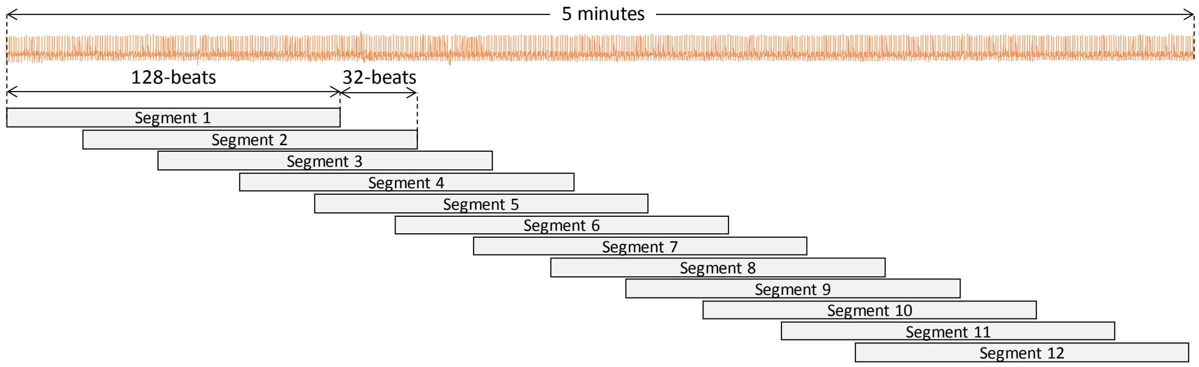

Figure 2. The 5-minutes electrocardiogram was divided into 128-beat-length segments overlapped 75% and proceeded by 32 bits

The 5-minutes ECG was divided into 128-beat-length segments. The segment was overlapped 75% and

proceeded by 32 beats in time axis. (Fig. 2) Microvolt T-wave alternans (mTWA) was calculated for each ECG segment. The mean of the mTWA of the segments was used for statistical analysis. The beat-to-beat intervals of all adjacent beat pairs in the segment were calculated. After converting the beat interval to heart rate, if the difference between the maximum and minimum value of the heart rate in one segment was more than 20, the corresponding segment was excluded.

ECG beat alignment

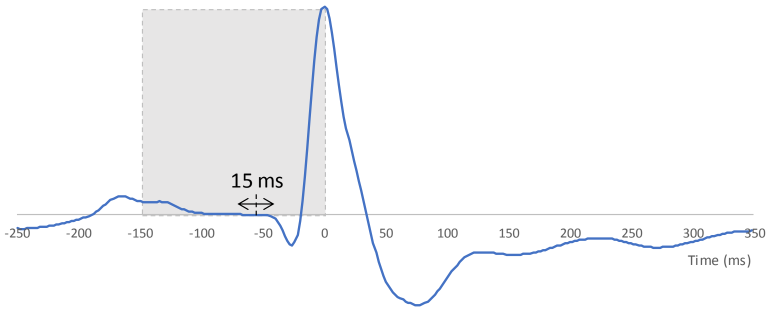

Figure 3. The baseline voltage was defined as the voltage at which the 15-ms window standard deviation was minimised.

The mean beats are obtained by averaging all beats in each ECG segment. The baseline voltage was defined as the voltage at which the 15-ms window standard deviation was minimised, and was searched within 150 milliseconds before the r-peak. (Fig. 3)

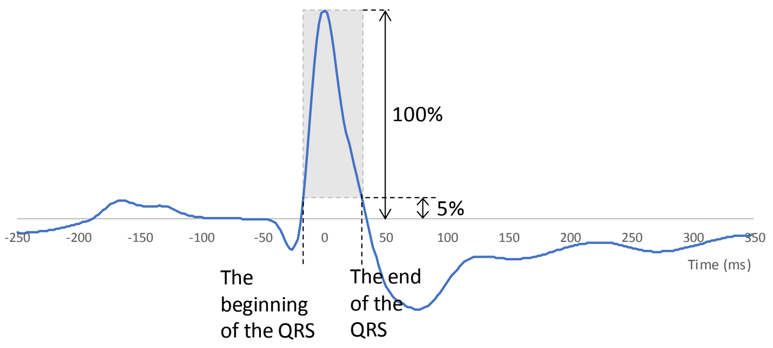

Figure 4. The QRS complex was defined as the time when the voltage was over the 5% of the R-peak voltage.

All beats were aligned to the average beat so that the correlation of the QRS interval was maximized. The beginning of the QRS complex was defined as the time when 5% of the R-peak voltage was achieved over the baseline voltage. The end of the QRS complex was defined as the time when the voltage returns to 5% of the R-peak voltage. Maximum movement for the alignment was limited to 6 milliseconds.

A beat with a correlation coefficient of 0.95 or less in the QRS complex was replaced with an average beat. If more than 25 beats were replaced, the corresponding segment was excluded.

Finally, all beats voltage levels were adjusted based on the peak voltage level of the average beat.

Power spectrums of 128 beats

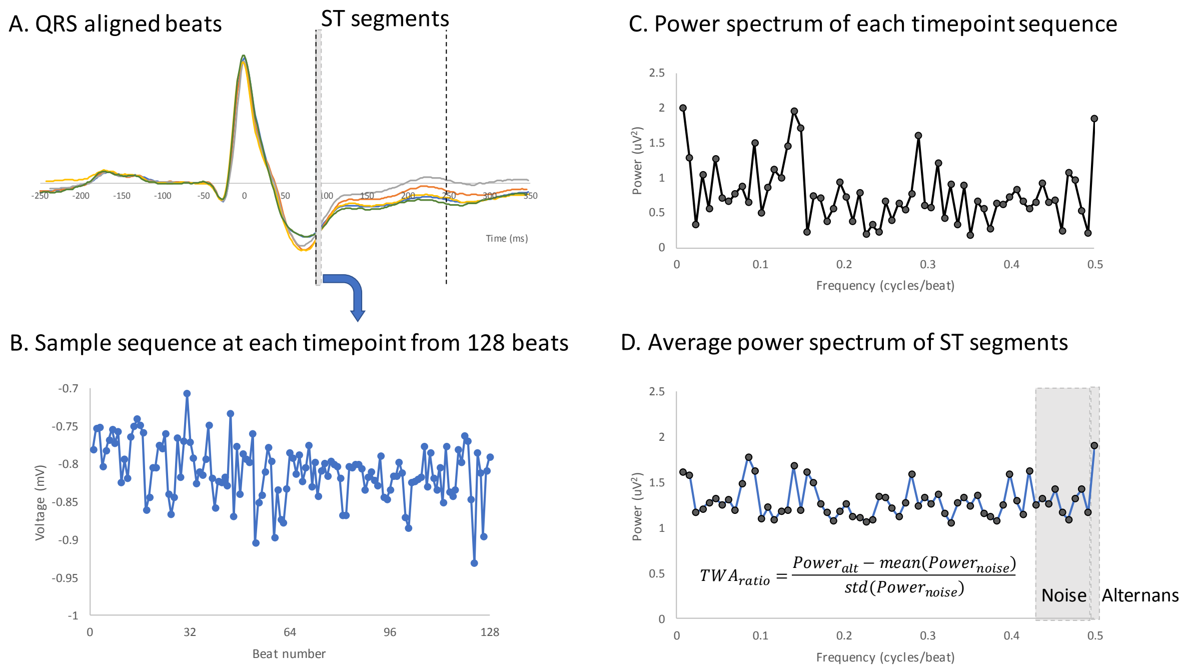

Figure 5. Power spectrums of 128 beats at each timepoint in ST segments were averaged

For each timepoint between 100 milliseconds and 250 milliseconds after R-peak, samples from the 128 beats were collected. (Fig. 5) Fourier transform was performed on this sequence to achieve power spectrum. The power corresponding to 0.44-0.49 beat was regarded as noise power. The alternans power was defined as the value obtained by subtracting the mean of the noise power from the power corresponding to 0.5 beat. The alternans ratio was defined as the alternans power divided by the standard deviation of the noise power. [3]

References

1. Pan J, Tompkins WJ. A real-time QRS detection algorithm. *IEEE Trans Biomed Eng* **3**, 230-236 (1985).

2. Meyer CR, Keiser HN. Electrocardiogram baseline noise estimation and removal using cubic splines and state-space computation techniques. *Comput Biomed Res* **10,** 459-470 (1977).

3. Richter S, Duray G, Hohnloser SH, How to analyze T-wave alternans. *Heart Rhythm* **2,** 1268-1271(2005).

Supplementary information 2. Definition of outcomes

1. Myocardial infarction

   Detection of cardiac biomarkers (troponin or CK-MB) more than 10 times the 99th percentile of the upper limit from a normal baseline with at least one of the following

   1) New left bundle branch block
   2) Developing of new pathological Q waves on the ECG
   3) Imaging evidence of new loss of viable myocardium or new regional wall motion abnormality
   4) Identification of a new intracoronary thrombus of the new graft or native coronary artery by angiography or autopsy

2. Renal failure

   KDIGO criteria

Stage	Serum creatinine	Urine output
1	1.5-1.9 x baseline or ≥ 0.3 mg/dl increase	< 0.5 ml/kg/h for 6-12 h
2	2.0-2.9 x baseline	< 0.5 ml/kg/h for > 12 h
3	3.0 x baseline, or increase in serum creatinine ≥ 4.0 mg/dl or initiation of RRT	< 0.3 ml/kg/h for ≥ 24 h or anuria for ≥ 12 h

Supplemental Table S1. Causes of in-hospital mortality.

Causes of death	In-hospital mortality	
	Positive (n=4)	Negative (n=3)
Multi-organ failure	2	0
Septic shock	1	2
Brain death	0	1
Unclear	1	0

Data are presented as the number
